# Vertigo in the Setting of COVID-19 Infection: A Case Report

**DOI:** 10.7759/cureus.34708

**Published:** 2023-02-06

**Authors:** Sanjana Devaragudi, Mohit Gupta

**Affiliations:** 1 Department of General Medicine, Apollo Institute of Medical Sciences and Research, Hyderabad, IND; 2 Department of General Medicine, Sharda Hospital, Greater Noida, IND

**Keywords:** covid-19, vertigo, vestibular neuritis, sars-cov-2, case report

## Abstract

Extensive research has been conducted on the pathophysiological sequelae of the SARS-CoV-2 virus. Newer symptoms pertaining to cardiovascular, gastrointestinal, and neurological systems, apart from the recognized respiratory system, are constantly being reported. We report a case of a 22-year-old female with a seven-day history of COVID-19 who presented to the emergency department with an acute vertigo attack associated with nausea and vomiting for three hours. Physical examination and audiometry showed no neurological and auditory deficits; she was diagnosed with vestibular neuritis. The patient was managed with betahistine hydrochloride and an antihistamine. There was a complete resolution of symptoms after seven days. There have been reports of new-onset vertigo associated with SARS-CoV-2 infection, but more well-designed studies are needed to establish an association between COVID-19 and vertigo.

## Introduction

Coronavirus disease 2019 (COVID-19) was declared a pandemic by the WHO in March 2020 [1]. SARS-CoV-2 chiefly affects the respiratory system, but neurological manifestations have also been widely reported. These include anosmia, headaches, dizziness, vertigo, encephalopathy, encephalitis, Guillain-Barré syndrome, meningitis, and stroke [2].

Vertigo is defined as a false sense of movement brought by the asymmetry in the vestibular system due to damage to the labyrinth, vestibular nerve, or central vestibular system of the brainstem or the cerebellum. Depending on the location of the pathology, it is classified as peripheral vertigo (vestibular components) or central vertigo (brain). Even though the relationship between SARS-CoV-2 and ear disorders is yet to be confirmed, a few authors have reported new-onset vertigo as a symptom or sequelae of COVID-19 [3-11]. Most cases were diagnosed with vestibular neuritis, cerebellitis, and intralabyrinthine hemorrhage and managed as per standard care along with treatment of the COVID-19 infection described by the national guidelines [12].

Vestibular neuritis is a self-limiting benign condition typically presenting with vertigo, nausea, vomiting, and gait abnormality. It is thought to be due to viral or post-viral inflammation of the vestibular component of the eighth cranial nerve. The diagnosis is made by tests such as the head impulse test, caloric test, smooth pursuit test, and vestibular evoked myogenic potentials [13]. In pure vestibular neuritis, the auditory function is preserved; when combined with unilateral hearing loss, it is called labyrinthitis [14]. The exact etiopathogenesis of vestibular neuritis and COVID-19 is not known. It is thought to be due to the hematogenous spread of the virus to the inner ear, due to the cytokine storm, or a consequence of the psychological/mental stress arising from the COVID-19 infection [2].

We discuss a case of a young female with new-onset vertigo after seven days of confirmed COVID-19 infection and having mild respiratory symptoms. The patient’s identification has been kept confidential in the case report.

## Case presentation

On February 8, 2022, a 22-year-old female presented to the emergency department with complaints of sudden onset, vertigo, nausea, and vomiting for three hours. She had no associated symptoms such as fever, earache, hearing loss, tinnitus, loss of limb movements, or speech impairment. She was not menstruating throughout her 14 days of illness. There was no abdominal pain/uterine cramps in this patient. The patient denied similar complaints in the past. She had no other comorbidities.

The patient had a history of COVID-19 infection seven days ago, which was confirmed via a nasopharyngeal swab for SARS-CoV-2 reverse transcription polymerase chain reaction (RT-PCR). The patient had a mild illness with symptoms such as intermittent fever, congestion, dry cough, and sore throat. This was treated at her home for seven days with acetaminophen 500mg, antihistamines (Cetrizine 10mg) as required, and a daily vitamin C. She did not take any antibiotics or anti-virals. Three doses of COVID-19 vaccine were taken previously. A family history of COVID-19 infection was present in her mother and father at the time of the patient being ill. However, they had no complaints of vertigo or any other symptoms after seven days of mild illness.

On arrival to the emergency room, the patient was afebrile. Her blood pressure was 100/60 mm of Hg, heart rate was 94 beats/minute, respiratory rate was 16 cycles/minute, and oxygen saturation showed 99% on room air. The patient was conscious and coherent. She was in severe distress due to vertigo, non-bilious, non-bloody vomiting, and dry heaving. She complained of a “sense of the room spinning.” She had no respiratory symptoms. Neurologically, she was cognizant, and cranial nerve examination was unremarkable. Gait could not be assessed as the patient was unable to sit up. Dix-Hallpike’s maneuver was deferred for the same reason. Nystagmus could not be elicited. Neuroimaging could not be performed as the patient was anxious and requested prompt management. A repeat RT-PCR for SARS-CoV-2 was not conducted as per the recommended guidelines at that time [15]. She was treated with IV fluids, an IV antiemetic (Ondansetron 8mg), and a betahistine hydrochloride 16mg tablet for vertigo with some improvement and was discharged.

**Figure 1:**
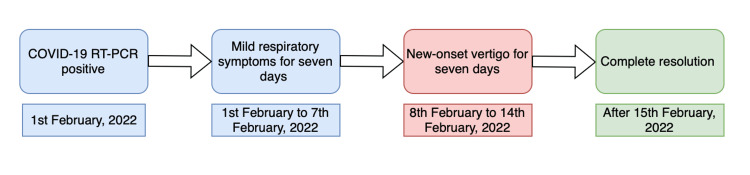
Timeline of presenting symptoms

She was evaluated the next day by a physician for persistent vertigo, which was not as severe as the previous day, a feeling of unsteadiness, and nausea. She was unable to tolerate food even after taking an antiemetic. There was a marked elevation in vertigo on rapid head movement to either side. On examination, nystagmus was not noted, and the head impulse test was negative, likely due to the two doses of betahistine hydrochloride the patient had taken before evaluation [13]. The patient could ambulate only with support. The patient did not report other neurological symptoms or signs such as dysarthria, dysphagia, limb weakness, a facial droop, headache, or stuttering, usually seen in a stroke/transient ischemic attack (TIA). Vertigo is also seen in cerebellar infarction and is usually accompanied by nystagmus, dysarthria, and truncal ataxia, which were not present in this patient. Benign paroxysmal positional vertigo (BPPV) was ruled out with a negative Dix-Hallpike’s test and a negative head roll test. A caloric test and vestibular evoked myogenic potential test could not be performed due to a lack of equipment at the facility. Pure tone audiometry showed no hearing loss in both ears, which ruled out Ménière’s disease. She denied any radiological investigations. Her lab reports were within normal limits (Table 1). A diagnosis of vestibular neuritis was made by excluding the differential diagnoses and based on the improvement shown on taking betahistine, an anti-vertigo medication. The patient was prescribed meclizine 25mg thrice daily, ondansetron 4mg as needed, and bed rest. There was a complete resolution of vertigo and nausea after seven days from the appearance of the first symptoms in the emergency (Figure 1). The patient was followed up every month for the next six months without any complaints of vertigo.

**Table 1:** Patient’s laboratory results RBC, red blood cell; TLC, total leukocyte count

Parameter	Result	Reference Range
Hemoglobin	13.2 g/dL	11.0-16.0
Hematocrit	39.2%	34.0-48.0
RBC count	4.71 million/uL	3.7-5.6
Platelet count	303,000/mm^3^	140,000-440,000
TLC	5,970/mm^3^	4,000-11,000
Neutrophils	62%	40-75
Lymphocytes	29%	20-45
Monocytes	7%	1-10
Eosinophils	2%	1-6
Basophils	0%	0-1
Erythrocyte sedimentation rate	20 mm/hr	0-20

## Discussion

Vertigo is an illusion of motion. It is defined by the Committee of Classification of vestibular disorders as the sensation of self-motion when none is occurring or as the sensation of distorted self-motion during an otherwise normal head movement [16]. It has been reported as a stand-alone symptom in patients with COVID-19 or as a post-COVID manifestation after a few weeks or months. Some significant differentials to consider when approaching a patient with vertigo in this scenario include BPPV, vestibular neuritis, Ménière’s disease, vestibular migraine, vertebrobasilar TIA, brainstem infarction, and cerebellar infarction, among others [12]. A case series published in 2022 discusses vestibular symptoms in patients with COVID-19, suggesting vestibular neuritis to be a diagnosis of exclusion when there are no associated alarming neurological symptoms, no abnormalities on imaging and blood tests, and negative inflammatory markers [4]. The clinical history and examination can not only distinguish between the causes but also help in triaging. In some cases, brain imaging and other tests such as electronystagmography, brainstem auditory evoked potential, and vestibular evoked myogenic potential may be ordered based on the likelihood of a grave disorder and the potential impact on treatment and prognosis [17].

Vestibular neuritis is generally thought to be a viral or post-viral inflammatory disorder affecting the vestibular portion of the eighth cranial nerve. It is characterized by an acute, spontaneous, peripheral vestibular ailment with rapid onset of severe vertigo, vomiting, nausea, and gait instability [13]. The pathophysiology of audio-vestibular symptoms in COVID-19 is unknown, but many theories have been proposed.

The central nervous system is thought to be affected via infiltration of the vascular structures by the virus, which enters through the angiotensin-converting enzyme-2 (ACE-2) receptors on the capillary endothelium. This vascular insult by virus particles makes access to the neural tissue and inner ear structures more convenient. The inner ear, highly vulnerable to ischemia, is affected by this virus-induced capillary damage and presents as hearing and balance disorders [2,4]. The cytokine storm associated with SARS-CoV-2 infection releases inflammatory mediators such as IL-6 and TNF alpha, which are thought to increase the binding of viral spike particles to the ACE-2 receptors. This increases the subsequent inflammation of the inner ear components [2,18]. Some studies state that vertigo could be a consequence of psychological and mental stress on the patient due to the diagnosis of COVID-19 and the required isolation. It could also be due to the re-activation of the latent herpes simplex virus in the vestibulocochlear nerve in response to stress [6,18]. Azithromycin, an antibiotic taken by many COVID-19 patients to treat respiratory symptoms, is an ototoxic drug that has also been considered a cause of the audio-vestibular effects seen in COVID-19 infection [2,18]. However, our patient had no history of ototoxic drug use.

The treatment for vestibular neuritis is supportive. Antiemetics, antihistamines, and benzodiazepines are commonly used. Patients should be started on lower doses with upward titration as needed. These medications suppress vestibular activity and are believed to impair central compensation; hence, long-term recovery is delayed. Corticosteroids are frequently used for refractory cases. A 10-day tapering dose of prednisone therapy is generally recommended in patients with no contraindications. Controlled trials have shown significant improvement in vestibular function with methylprednisolone at 12-month follow-up [19]. Vestibular rehabilitation is often recommended, with most results seen in patients referred to a vestibular physical therapy evaluation and completing an individually designed program [13,20]. Our patient states that although there was an improvement in the severity of vertigo on using meclizine, it persisted throughout the day for seven days. There was a complete cessation of vertigo after seven days. Hence, there was no requirement for corticosteroids.

Currently, there are no guidelines for treating vertigo in the setting of COVID-19. However, the use of corticosteroids for vestibular neuritis in COVID-19 has been described in some cases. A 10-day course of oral prednisolone or IV steroids was given. The dose ranged from 20mg to 60mg, which was tapered. Some were treated with antihistamines or vestibular rehabilitation only. The symptoms lasted anywhere between 10 days and 10 weeks [4-10].

Limitations

The lack of repeat RT-PCR testing for SARS-CoV-2 at the onset and/or after the resolution of vertigo could not help establish a complete association with COVID-19. Neuroimaging is strongly indicated to rule out alternative diagnoses in addition to clinical evaluation, but it could not be done in this patient due to refusal.

## Conclusions

This case adds to the current literature on COVID-19 disease and vertigo. It emphasizes the importance of atypical symptoms presenting during the infection course or after the cessation of ongoing symptoms. The cause of vestibular neuritis seen in this patient could be either the direct impact of SARS-CoV-2 on inner ear structures or the stress. Physicians should inform patients of such, albeit rare, consequences of COVID-19 and educate them about its management. More evidence and research are needed to understand the relationship between COVID-19 and vestibular symptoms, and guidelines on the treatment of vertigo and vestibular neuritis in COVID-19 patients are required.
